# Pharmaceutical Functionalization of Monomeric Ionic Liquid for the Preparation of Ionic Graft Polymer Conjugates

**DOI:** 10.3390/ijms232314731

**Published:** 2022-11-25

**Authors:** Aleksy Mazur, Katarzyna Niesyto, Dorota Neugebauer

**Affiliations:** Department of Physical Chemistry and Technology of Polymers, Faculty of Chemistry, Silesian University of Technology, 44-100 Gliwice, Poland

**Keywords:** dual drug systems, ionic conjugates, graft copolymers, co-delivery systems

## Abstract

Polymerizable choline-based ionic liquid (IL), i.e., [2-(methacryloyloxy)ethyl]-trimethylammonium (TMAMA/Cl¯), was functionalized by an ion exchange reaction with pharmaceutical anions, i.e., cloxacillin (CLX¯) and fusidate (FUS¯), as the antibacterial agents. The modified biocompatible IL monomers (TMAMA/CLX¯, TMAMA/FUS¯) were copolymerized with methyl methacrylate (MMA) to prepare the graft copolymers (19–50 mol% of TMAMA units) serving as the drug (co)delivery systems. The in vitro drug release, which was driven by the exchange reaction of the pharmaceutical anions to phosphate ones in PBS medium, was observed for 44% of CLX¯ (2.7 μg/mL) and 53% of FUS¯ (3.6 μg/mL) in the single systems. Similar amounts of released drugs were detected for the dual system, i.e., 41% of CLX¯ (2.2 μg/mL) and 33% of FUS¯ (2.0 μg/mL). The investigated drug ionic polymer conjugates were examined for their cytotoxicity by MTT test, showing a low toxic effect against human bronchial epithelial cells (BEAS-2B) and normal human dermal fibroblasts (NHDF) as the normal cell lines. The satisfactory drug contents and the release profiles attained for the well-defined graft polymers with ionically bonded pharmaceuticals in the side chains make them promising drug carriers in both separate and combined drug delivery systems.

## 1. Introduction

The delivery of active pharmaceutical ingredients (API) is one of the most actual research trends, since the majority of a drug’s action is usually limited by many factors, including the low solubility of the drug, which reduces its bioavailability [1]. The hydrophilicity of drugs can be improved by encapsulation with amphiphilic copolymers [2] or by conjugation with hydrophilic species/polymers [3,4]. Another possibility is drug conversion into salt by biofunctionalization of ionic liquids (ILs) [5,6]. ILs are salts in a liquid state below 100 °C with unique properties, such as high thermal and chemical stability, solubility in many solvents, very often low-toxicity, and an ability to be used in a variety of modifications to generate proper activity for the required applications [7,8,9]. Moreover, their biological properties, including antibacterial, local anesthetic, antifungal, anti-acne, and antibiotic actions, mean ILs are often used in medicine [10,11]. The API-IL combination allows the tuning of the APIs’ properties from solid into salts in a liquid state at room or body temperature. These are known as API-ILs, which have radically better absorbability and stability, as well as permeability in comparison to their solid analogs [12,13,14]. The drug connection with ILs through ionic bonding is a convenient way to introduce the pharmaceutical ions [15]. The important criterion that is required in the use of ILs as matrices for drug delivery is their toxicity and biocompatibility. Biocompatible ILs carriers based on imidazolium, phosphonium, lidocaine, ammonium, pyridinium, and choline cations have been biofunctionalized with various active pharmaceutical ions, including sulfasalazine [13], amoxicillin, penicillin-G [16], ibuprofen [17,18], ampicillin [19], salicylate [20] counterions, and the derivatives of nalidixic, niflumic, pyrazinic and picolinic acid [21] have been tested.

The use of polymerizable (monomeric) ILs allows the obtainment of high-molecular carriers based on poly(ionic liquid)s (PILs) [22]. Most of the systems based on linear API-PILs have been already synthesized as a result of the polymer post-modification by introducing anions of anti-inflammatory drugs, including antibiotics, e.g., ampicillin [23] and sulfacetamide [24] anions. In turn, the grafted PILs have been employed for salicylate [25], *p*-aminosalicylate, clavulanate [26], piperacillin, and fusidate [27] delivery. The latter topology has ensured an extended period of drug release and increased stability of the carrier.

The preliminary designing of drug delivery systems provides the possibility of adjusting their physicochemical properties. There are many pathways for obtaining structures which are available to attach more than one API and can be used to prepare drug co-delivery systems with guaranteed synergistic drug action and improved treatment efficacy [28,29,30]. The dual drug systems are usually used in cancer treatment or diseases caused by drug-resistant strains, where APIs are conjugated and/or encapsulated. Co-delivery systems have been reported for the ionic conjugates based on linear amphiphilic PILs to deliver anti-inflammatory APIs, i.e., sulfacetamide or salicylate in the ionic form, and encapsulated non-ionic erythromycin, indometacin, or quercetin [24]. The strategy of combining two types of drug matrix binding has also been used in analogous systems based on grafted PILs, which were used for the transport of antibacterial drugs with anti-tuberculosis activity, i.e., ionic fusidate and non-ionic rifampicin [31]. In general, the drug-polymer conjugates exhibit longer plasma circulation times compared to micelles with encapsulated drugs whose persistence depends on critical micelle concentration, and therefore, they are less stable when diluted in the bloodstream [32,33]. However, drug conjugation by covalent bonds forms more stable systems requiring hydrolytic cleavage to release the drug, whereas in the ionic conjugates, this occurs by ionic exchange.

Here we report the new biofunctionalized choline monomeric ILs, which were prepared from the commercially available [2-(methacryloyloxy)ethyl]trimethylammonium chloride (TMAMA/Cl¯) through the ionic exchange of a chloride counterion to a pharmaceutical one. The drugs used in this study, i.e., cloxacillin (CLX¯) or/and fusidate (FUS¯), were chosen as antibacterial agents, which are known from their synergistic action [34]. CLX¯ is a type of penicillin used against staphylococcal, pneumococcal, and beta-hemolytic streptococcal infections, however, it is not effective for the treatment of methicillin-resistant *Staphylococcus aureus* [35]. FUS¯ is active against staphylococci, beta-hemolytic streptococci, and also against most *Corynebacterium* or *Clostridium* strains [36]. Furthermore, it is also used to successfully treat *Staphylococcus aureus,* but has significantly lower activity against *S. aureus* in comparison to CLX¯ [37]. Therefore, both pharmaceutics are suitable candidates to be used in either individual or combination therapies. The formulation containing both cloxacillin and fusidate is not commercially available, however it is used in the same therapy as the separate medicines, e.g., CLX as *Cloxapen, *Cloxacap*, *Tegopen, Orbenin,** or *Syntarpen*, orand FUS as Fucidin^®^, due to their satisfactory co-activities, as mentioned above. In our studies, the monomers of TMAMA with pharmaceutical counterions were used in the atom radical transfer polymerization (ATRP) via the “grafting from” technique to accomplish the well-defined graft copolymers. Depending on the approach, the graft copolymers were designed as single drug systems bearing CLX¯ or FUS¯ (by copolymerization of proper TMAMA with methyl methacrylate (MMA)) and as dual drug systems carrying CLX¯ and FUS¯ (by terpolymerization of both TMAMA monomers and MMA). In vitro drug release studies in PBS (37 °C) were carried out. In this study, the influence of a co-ion on the degree of polymerization and the drug release efficiency were investigated.

## 2. Results and Discussion

**Figure 1:**
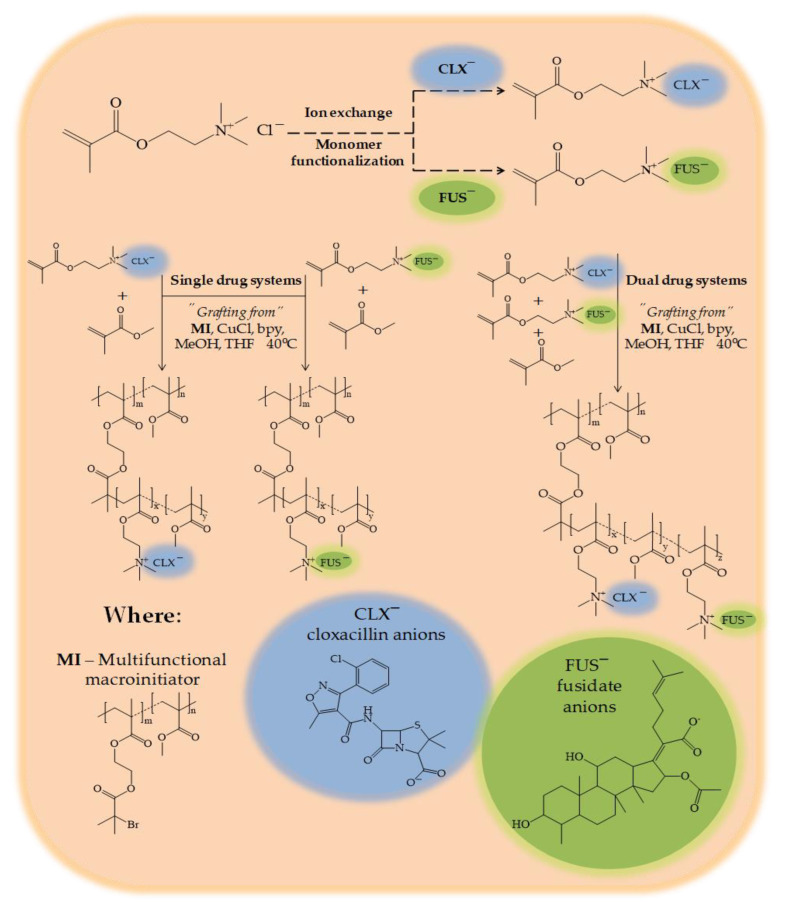
Strategy for obtaining drug carriers through successive ion exchange reactions onto a TMAMA/A¯ monomer, and the synthesis of ionic copolymers with the pharmaceutical anions A¯.

The presence of a chloride counterion in the polymerizable ionic liquid (IL), i.e., [2-(methacryloyloxy)ethyl]trimethylammonium chloride (TMAMA/Cl), was convenient for the ion exchange reaction to introduce pharmaceutical anions. For this purpose, the fusidic acid sodium salt (NaFUS) or cloxacillin sodium monohydrate (NaCLX) were used. As a result, the monomeric ILs functionalized with pharmaceutical anions, i.e., [2-(methacryloyloxy)ethyl]trimethylammonium cloxacillin (TMAMA/CLX*¯*) and [2-(methacryloyloxy)ethyl]trimethylammonium fusidate (TMAMA/FUS*¯*) were obtained (Figure 1).

**Figure 2:**
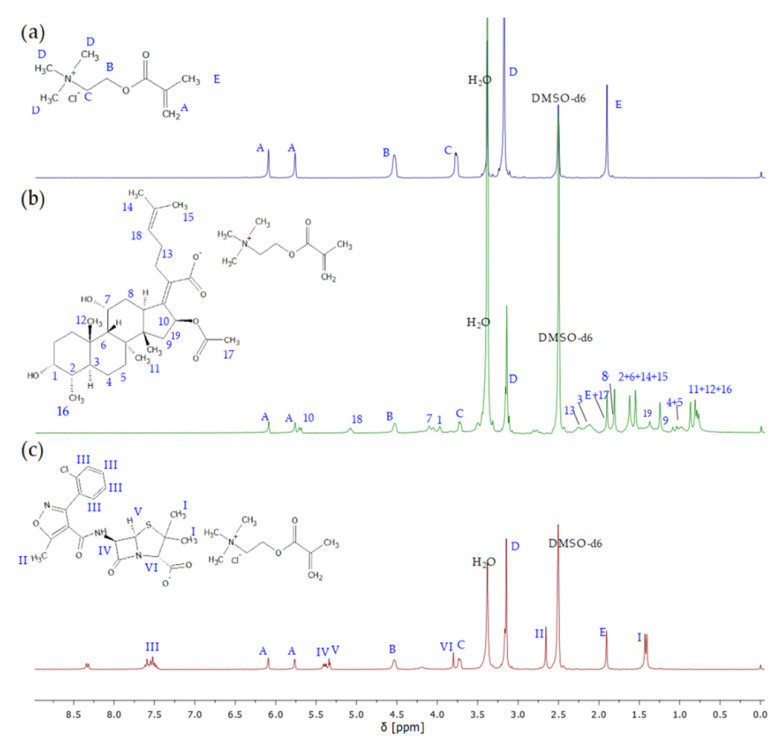
^1^H NMR spectra before ionic exchange of TMAMA/Cl¯ (**a**) and after modification for TMAMA/FUS¯ (**b**) and TMAMA/CLX¯ (**c**).

The structures of IL monomers were confirmed by ^1^H NMR (Figure 2) and FT-IR (Figure S1) spectra showing characteristic proton signals and absorption bands which were assigned to those from TMAMA and proper pharmaceutic counterions, as the product from the initial substrates TMAMA/Cl and pharmaceutical sodium salt. The most distinctive signals correspond to the vinyl group A at 5.7–6.2 ppm (900–1500 cm^−1^) and the ammonium cation D at 3.1–3.27 ppm (1320–1370 cm^−1^) regarding TMAMA units; in turn, the representative signals at 5.0–5.15 ppm (1550 cm^−1^) and 7.5–7.7 ppm (1590 and 1480 cm^−1^) came from the aromatic ring in CLX¯ (III in Figure 2c) and -CH = group in FUS¯(18 in Figure 2b), respectively. Additionally, the ^1^H NMR spectra were used to evaluate the efficiency of ion exchange, comparing the integration of signals in the range of 3.11–3.23 ppm assigned to the protons in trimethylammonium groups (D) before and after drug introduction (Figure S3). The similar degrees of ion exchange (71 and 74% regarding to TMAMA/FUS¯ and TMAMA/CLX¯, respectively) indicated that the ionic conjugation of pharmaceutical anions was not affected by the drug nature.

The well-defined graft copolymers based on biologically active choline species with pharmaceutically active counterions, i.e., CLX¯ and/or FUS¯, were synthesized directly by atom transfer radical polymerization (ATRP) at 40 °C. The reaction was catalyzed by the CuCl/bpy complex using the solvent mixture of methanol and tetrahydrofuran (MeOH/ THF). The multifunctional macroinitiator (MI), which was a copolymer composed of methyl methacrylate and 2-(2-bromoisobutyryloxy)ethyl methacrylate (P(MMA-*co*-BIEM) with a degree of polymerization (DP_n_) equal to 165), contained 29% of bromoester initiating groups, which were used for the formation of side chains. The first route involved the copolymerization of the ionic monomer, i.e., TMAMA/CLX¯ or TMAMA/FUS¯, with the non-ionic MMA in the molar ratio of 25:75 to achieve the side chains carrying one type of pharmaceutic (P(TMAMA/FUS¯-*co*-MMA) or P(TMAMA/CLX¯-*co*-MMA) as the single drug systems). The second path was based on the terpolymerization of both ionic monomers with CLX¯ and FUS¯ counterions, and MMA in the ratio equal to 12.5:12.5:75. The ionic conjugate P(TMAMA/CLX¯-*co*-TMAMA/ FUS¯-*co*-MMA) was designed to be capable of transporting two drugs at the same time (the dual drug system). In all cases, the grafting degree (DG) of polymers adjusted by the content of initiating groups was equal to 29 mol %, which corresponded to 48 grafts as the number of side chains (n_sc_) defined by the number of repeating BIEM units in the main chain. The strategy of functionalization of IL monomers (pre-polymerization modification) and their usage instead of the TMAMA/Cl¯ one for the graft copolymer synthesis allows for skipping the necessity of polymer modification, in which yield is highly dependent on the efficiency of an ion exchange of chloride anions in the polymer. In the presented strategy, the drug content is mainly affected by the relative reactivity of ionic monomer(s) to nonionic one(s), as well as by monomer conversion, which also defined the length of side chains (DP_sc_). The characteristics of graft copolymers are summarized in Table 1.

**Figure 3:**
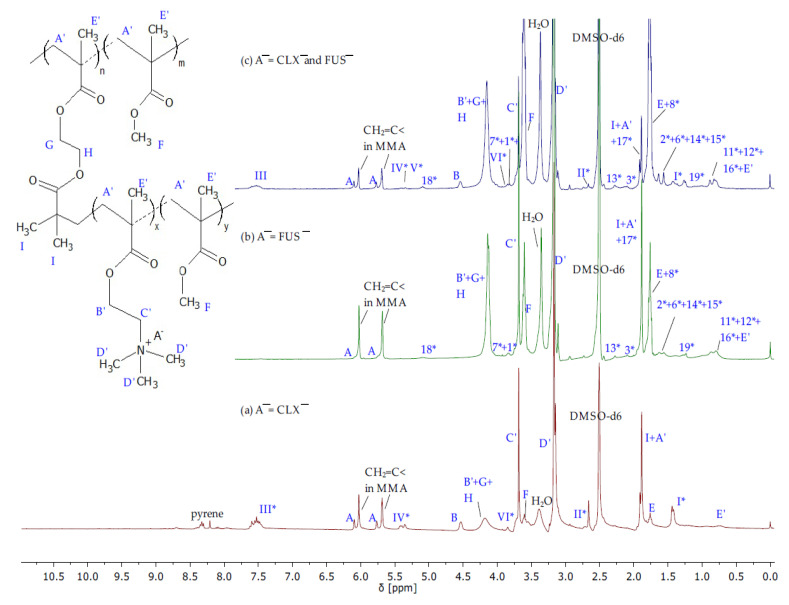
^1^H NMR spectra of reaction mixtures during formation of polymers carrying (**a**) CLX¯, (**b**) FUS¯, and (**c**) both CLX¯ and FUS¯ anions, where the signals coming from pharmaceutical anions are denoted using the symbol *.

TMAMA/CLX¯ was well soluble in MeOH, thus the ratio of MeOH:TMAMA/CLX¯ = 1:1 was sufficient to reach 33% of IL monomer conversion after 2 h. In the case of TMAMA/FUS¯, copolymerization an increased amount of polar co-solvent was required due to the stickiness of monomeric IL. These tuned conditions provided slightly reduced conversion of IL monomer yielding 50% within 4 h, which can be also explained by the risk of transesterification of TMAMA to MMA activated by a larger amount of methanol, as it was declared earlier in the case of polymerization of TMAMA/Cl [38]. Relating to G_CLX¯/FUS¯ obtained at the initial molar ratio of TMAMA/CLX¯:TMAMA/FUS¯ = 1:1, the total conversion of IL monomers was the lowest (24%) in comparison to previous reactions incorporating a single monomeric IL because of the wide-ranging drug–drug steric and electrostatic interactions. Finally, the obtained ionic graft copolymers varied with the content of IL fraction (F_TMAMA_ = 19 vs. 50 vs. 29 mol.% in G_TMAMA/CLX¯ vs. G_TMAMA/FUS¯ vs. G_TMAMA/CLX¯/ FUS¯, respectively). The formation of side chains bearing ionic drugs was confirmed by ^1^H NMR spectra (Figure 3) showing the signals related to FUS¯ and CLX¯, which were observed earlier on the spectra of corresponding monomers (Figure 2). Additionally, the signals C’ and D’ at 3.64–3.78 ppm and 3.0–3.27 ppm coming from protons of -CH_2_-N^+^- and -N^+^- (CH_3_)_3_ groups were present, whereas the intensities of signals A’ and E’ from methylene and the methyl group in the polymethacrylate chain, and F’ from the methoxy group of MMA units were significantly increased. The monomer conversions were determined on the base of the reaction mixture spectrum by estimating the integration of signals for unreacted TMAMA (6.07 ppm) and MMA (6.02 ppm) in relation to the constant intensity of the pyrene signal (8.19–8.22 ppm) as the internal standard. The analysis of a purified and dried sample of G_CLX¯/FUS¯ (Figure S4) was required to evaluate the individual content of TMAMA/CLX¯ and TMAMA/FUS¯ in the dual drug copolymer. For this purpose, the integration of signals I* (6 protons in CLX¯) and 10* (1 protons in FUS¯) enabled the determination of the ratio of CLX¯/FUS¯ in TMAMA units (56/44) and then the calculation of the individual number of repeating units TMAMA/CLX¯ (DP_TMAMA/CLX¯_ = 3) and TMAMA/FUS¯ (DP_TMAMA/FUS¯_ = 3). This TMAMA ratio with a slight domination of units containing CLX¯ in the double drug polymer was very well correlated with the tendency of X_TMAMA/CLX¯_ ≥ X_TMAMA/FUS¯_ observed for syntheses of the single drug polymers, which could suggest lower steric repulsion of TMAMA/CLX¯.

The fraction of ionic units (F_TMAMA_) and the polymer molecular weight (M_n_) were calculated using monomer conversion (Table 1). However, the M_n_ values were significantly higher than that estimated by GPC, because the latter measurements were performed with the use of the linear polystyrene calibration, yielding apparent values. The dispersity indices of the single drug polymers were relatively low (Ð = 1.4–1.5) and even the excess of MeOH did not reduce the control of polymerization G_FUS¯, maintaining the homogeneity of polymers with similar molecular weight distribution. It has to be mentioned that the incorporation of monomeric IL can generate steric hindrance and decrease the access to growing active sites. Moreover, due to possible coordination of anions to the catalyst, increased polarity of sample, and also the slower reaction, the system with prevailing MMA units could promote occurrence of the side reactions. Comparing the polymers carrying one type of anion, the combination of repeating units with two different pharmaceutical anions in the side chains magnified this problem, providing higher dispersity of the double drug polymer G_CLX¯/FUS¯ (Ð = 1.9).

The obtained graft copolymers containing ionically conjugated pharmaceutical anions, i.e., G_CLX¯ and G_FUS¯, served as matrices of single drug delivery systems, whereas G_CLX¯/FUS¯ was designed as a dual drug system for co-delivery. Choline cations are useful for the increase in bioavailability of FUS¯ and CLX¯ anions, as well as their potential permeation through the plasma membrane. Copolymers varied by the type of anion, i.e., FUS¯ or CLX¯, which are commonly used as an antibiotics in the treatment of the, e.g., skin, eye, blood, bone, and lung infections. In general, the drug content (DC), which was evaluated by UV–Vis, increased with TMAMA content in the single systems, but it could be also affected by the type of drug anion (Table 2). Due to the more complex structure of the dual drug system, where the two methacrylate IL units with different drug anions were introduced, as well as their steric hindrance and various interactions between the anions, i.e., CLX-CLX, FUS-FUS, FUS-CLX, the DC values of both anions in G_CLX¯/FUS¯ was lower compared to DC in G_CLX¯ and G_FUS¯ (CLX¯: 41 vs. 44% and FUS¯: 33 vs. 53%, respectively).

**Figure 4:**
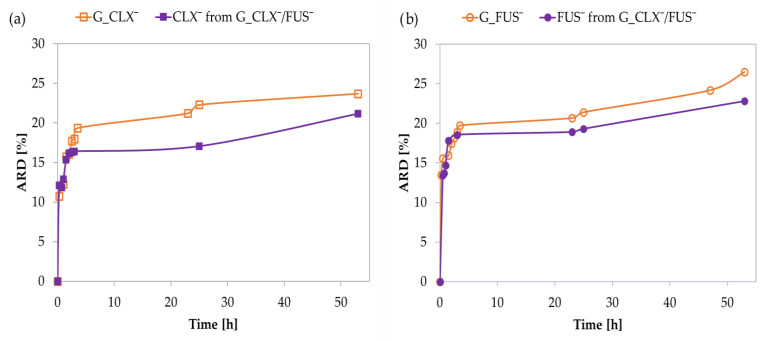
Kinetic release profiles of (**a**) CLX¯ from single drug system G_CLX¯ and dual-drug system G_CLX¯/FUS¯ and (**b**) FUS¯ from single drug system G_FUS¯ and dual-drug system G_CLX¯/FUS¯ based on PIL graft copolymers.

In vitro drug release studies were performed in PBS at 37 °C via the dialysis method. Single systems demonstrated an initial burst effect, and 15% of drug anions were exchanged with phosphate ones in a buffer solution within 0.5–1 h, and then further release was noticeably slower, yielding an additional 9–12% of drug up to 50 h (Figure 4). The kinetic release profiles for CLX¯ and FUS¯ by the dual co-delivery system were similar to the above-described, showing rapid release of 15% of both pharmaceutical anions up to 45 min. After this time, the release slowed down, and finally 24% of the drug was released within 50 h. The interactions between CLX¯ and FUS¯ did not significantly limit their release, compared to the single drug systems, where the amounts of released drugs (ARD) were slightly higher than in the dual drug system (CLX¯: 24 vs. 21% and FUS¯: 27 vs. 23%, respectively) (Table 2). In relation to the DC values, the final concentrations of released drugs (CRD) from G_CLX¯/FUS¯ were lower than those for G_CLX¯ and G_FUS¯. Comparing DC and ARD values in single systems, it can be concluded that the type of anion is a more important factor influencing the amount of introduced and released drug than F_TMAMA,_ which was more than twice as large in the G_FUS¯ than in the G_CLX¯. Regardless, it seems that single systems with simultaneously higher F_TMAMA_ and lower DP_sc_ tend to have higher DC, whereas ARD appears to be independent of conjugate characteristics.

The previously described ion exchange reaction onto the chloride choline-based graft polymers [24] can be incomplete, providing the presence of pharmaceutical anions and the unchanged Cl¯, which can reduce the drug content. The reported post-polymerization modified copolymers bearing FUS¯ (called as the reported FUS systems) with similar DG (26 mol%) were used to produce single (DC = 52% ARD = 53%, CRD = 7.2 μg/mL) and dual ionic–nonionic (DC = 55% ARD = 31%, CRD = 4.3 μg/mL) drug systems. However, the side chains were longer (DP_sc_= 35) and F_TMAMA_ = 39 mol%. It can be seen that in comparing the reported FUS single system and G_FUS¯ carrier with shorter side chains (DP_sc_ = 26) and higher F_TMAMA_ = 50 mol%, the DC value was the same, but ARD from G_FUS¯ was twice as low.

On the other side, the release data for the reported FUS dual system were close to that obtained for G_FUS¯, but they were higher (including DC value) than in case of the G_CLX¯/FUS¯ dual system. It can be assumed that the latter one was characterized by stronger interactions between both ionic drugs with a polymer matrix, whereas the possible limitation caused by the steric hindrance of drug species in the TMAMA monomers used in terpolymerization can be responsible for lower DC.

**Figure 5:**
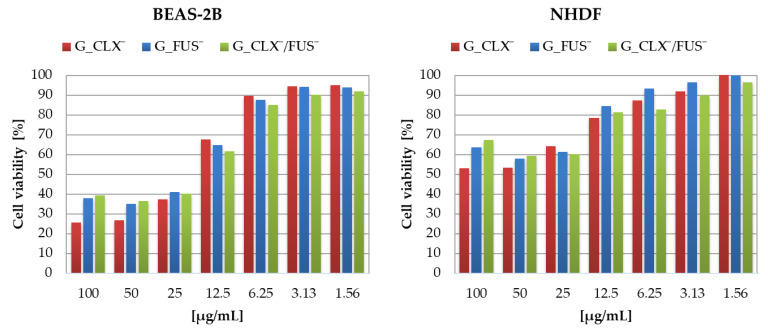
Viability of NHDF and BEAS-2B cell lines treated by graft copolymers at different concentrations after 72 h of incubation.

**Figure 6:**
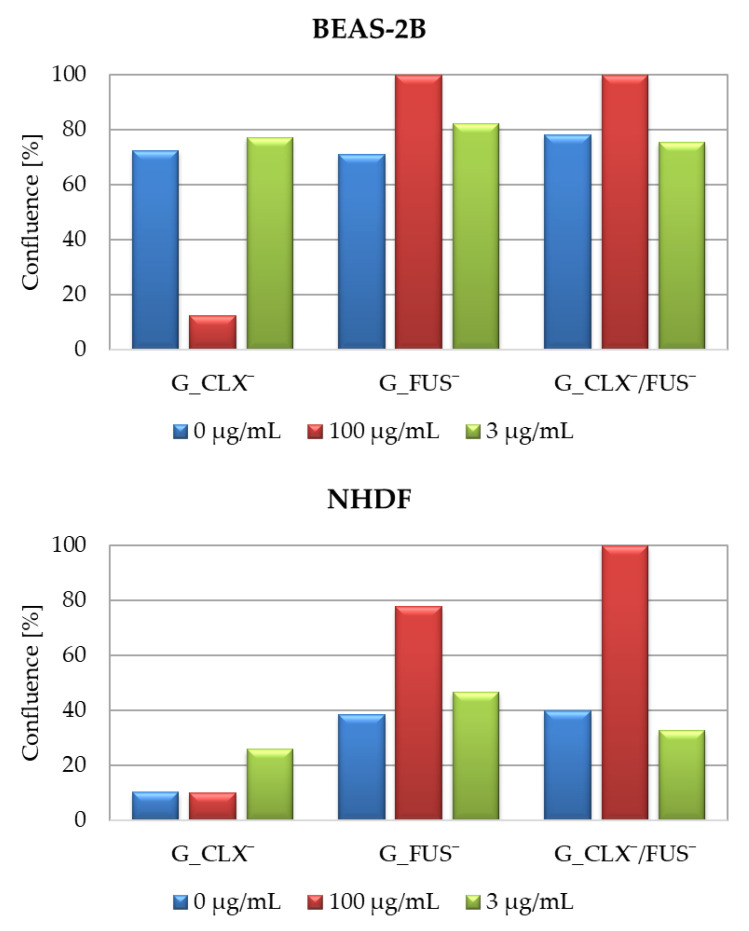
The confluence of cells treated by ionic conjugates in comparison to untreated cells.

**Figure 7:**
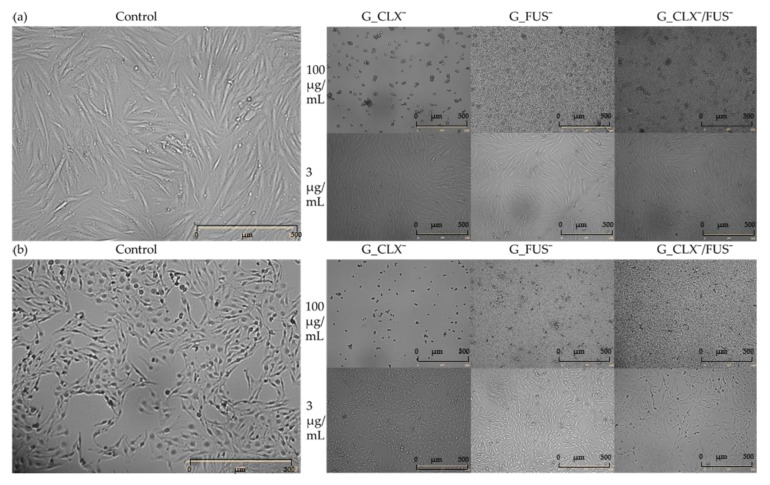
Microscopic images by Live Cell Analyzer for control vs. treated (**a**) NHDF (**b**) BEAS-2B cells by G_CLX¯ G_FUS¯ and G_CLX¯/FUS¯ at 100 and 3 µg/mL.

The designed drug carriers were evaluated by biological studies, where the cytotoxic activities of copolymers were analyzed against human bronchial epithelial cells (BEAS-2B) and normal human dermal fibroblasts (NHDF). The cell viabilities determined by MTT test were measured at the series concentrations of IL choline-based copolymer solutions (1.56–100 μg/mL) (Figure 5). The proliferation in both cell lines was not significantly inhibited, since at 100 μg/mL of each investigated polymer mixture (containing approximately 0.2–0.42 μg/mL of drug), the cell viability was not lower than 20% compared to untreated controls representing 100%. Viabilities of cells BEAS-2B treated by G_CLX¯ and G_FUS¯ solutions ranged from 26 to 95%, and from 39 to 92% when G_CLX¯/FUS¯ was administrated. Drug delivery systems indicated a lower toxic effect towards NHDF cells, where viability ranged from 53–100% (single drug systems) and 67–97% (dual drug system). In general, cytotoxicity decreased with lowering concentrations of polymer. However, it can be seen that at a higher concentration (100 µg/mL) the cells’ proliferation was slightly improved in the presence of FUS¯ compared to 25–50 µg/mL due to cell adaptation [39], whereas in front of CLX¯, it was nearly at the same level. Moreover, after 72h of incubation, the confluence, which is a percentage of covered surface by the cells, was evaluated for untreated control cells and conjugate samples at concentrations 100 µg/mL and 3 µg/mL (Figure 6). For both cell lines, in the case of carriers bearing FUS¯, generally an increase of confluence compared to controls is also demonstrated on the microscopic images (Figure 7). It can be noted that exposure of NHDF cell lines to G_FUS¯ or G_CLX¯/FUS¯ caused a significant increase in confluence at 100 µg/mL with respect to controls, and crowding led to the cell death in comparison to BEAS-2B cells, where the increase was not that high. Regarding the cells treated by CLX¯, the reproduction and activity of BEAS-2B cells was greatly reduced at 100 µg/mL in comparison to untreated cells, whereas in case of NHDF cells, confluence was not affected. Additionally, for both cell lines at 3 µg/mL, cell occupied area was slightly raised. Exposure of the cell lines to dual drug systems caused a slight decrease in confluence at 3 µg/mL, whereas at 100 µg/mL it was higher, especially in the case of NHDF cells, where it increased by approx. 40%.

## 3. Materials and Methods

Methyl methacrylate (MMA, Alfa Aesar, Warsaw, Poland) was dried using molecular sieves (type 4A, bulk density 640–670 kg/m^3^, Chempur, Piekary Śląskie, Poland). [2-(Methacryloyloxy)ethyl]trimethylammonium chloride (TMAMA/Cl, 80% aq. solution, Sigma-Aldrich, Poznan, Poland) was concentrated under vacuum to a solid product. Copper(I) chloride (CuCl, Fluka, 98%, Steinheim, Germany) was purified by stirring in glacial acetic acid, followed by filtration and washing with ethanol and diethyl ether, then dried under vacuum. Methanol (MeOH) and diethyl ether were obtained from Chempur (Piekary Śląskie, Poland). Cloxacillin sodium monohydrate (NaCLX) and fusidic acid sodium salt (NaFUS) were purchased from Alfa Aesar (Warsaw, Poland) and used without prior purification. Phosphate-buffered saline (PBS, pH = 7.4), 2,2′-bipyridine (bpy), tetrahydrofuran (THF), DMEM-F12 medium, 3-(4,5-dimethylthiazol-2-yl)-2,5- diphenyltetrazolium bromide (MTT), and trypsin were obtained from Sigma-Aldrich (Poznań, Poland). Physiological phosphate buffered saline (PBS without Ca and Mg, PAN-Biotech Gmbh, Aidenbach, Germany), fetal bovine serum (FBS, EURx, Gdańsk, Poland), and Annexin-V binding buffer (BD Biosciences, San Jose, CA, USA) were used without prior preparation. Human bronchial epithelial cells (BEAS-2B) and normal human dermal fibroblasts (NHDF) were purchased from ATCC (Cat# ATCC^®^CRL-9609; Manassas, VA, USA). The multifunctional macroinitiator (MI), which is a copolymer composed of methyl methacrylate and 2-(2-bromoisobutyryloxy)ethyl methacrylate (P(MMA-*co*-BIEM)), was synthesized as described previously [26].

### 3.1. Ion Exchange of TMAMA/Cl to TMAMA/A¯

NaFUS (508.76 mg, 0.94 mmol) and dry TMAMA/Cl (196.16 mg, 0.94 mmol) were separately dissolved in MeOH (2.55 mL and 0.98 mL, respectively) (TMAMA/Cl: MeOH = 1:5 *w/v*). Next, a drug solution was added dropwise to a monomer one. During drug addition, the mixture was constantly stirred for 2 h during the ion exchange reaction. After NaCl salt precipitation, the crystals were filtered and washed twice using 1 mL methanol. Then, the solvent was evaporated leaving the monomer with introduced pharmaceutical anion, i.e., TMAMA/FUS¯, which was dried under the vacuum (686.81 mg).

The analogical procedure was applied for the preparation of TMAMA/CLX¯ (992.60 mg) using the equimolar ratio of TMAMA/Cl (355.11 mg, 1.71 mmol) and CLX sodium salt (813.60 mg, 1.71 mmol).

The efficiency of ion exchange (75% and 71% for FUS¯ and CLX¯, respectively) was evaluated by ^1^H NMR analysis of the received monomers.

^1^H NMR data (DMSO-d_6_, δ, ppm): 1.7–1.9 (3H, -CH_3_), 3.1–3.27 (9H, -N^+^(CH_3_)_3_), 3.7–3.8 (2H, -CH_2_-N^+^-), 4.4–4.6 (2H, -CH_2_-O-), 5.7–6.2 (2H, =CH_2_); FUS¯: 5.65–5.72 (1H, >CH-O-), 5.0–5.15 (1H, =CH-), 4.0–4.11 (2H, >CH-OH), 3.9–4.0, 2.0–2.2, 1.58–1.68 (1H, >CH-), 2.2–2.3, 1.77–1.88, 1.35–1.4, 0.93–1.11, (2H, -CH_2_-), 1.86–1.97, 1.6–1.68, 1.2–1.3, 0.74–0.9 (3H, -CH_3_); CLX¯: 7.5–7.7 (4H, aromatic ring) 5.38–5.43 (1H, >CH-NH-), 5.25–5.34 (1H, >CH-S-), 3.67–3.75 (1H, >CH-N-), 2.6–2.7, and 1.35–1.46 (9H, -CH_3_).

FT-IR data (Figure S1): C=C vinyl (900–1050 cm^−1^), C-O (1150–1260 cm^−1^), C-H (1430–1490 cm^−1^), C-N (1635 cm^−1^), C=O (1720 cm^−1^), C-H (2860–2930 cm^−1^); FUS¯: C-H (2860–2930 cm^−1^), C=C (1550 cm^−1^, 3030 cm^−1^) and =C< (1375 cm^−1^); CLX¯: C=O (1715 cm^−1^), C=N (1670 cm^−1^), C=C aromatic (1590 and 1480 cm^−1^), C-Cl (770 cm^−1^); O-H in FUS¯, N-H in CLX¯, and moisture (3100–3500 cm^−1^).

### 3.2. Synthesis of Polymers

#### 3.2.1. Ionic Graft Copolymers Bearing CLX¯

The comonomers TMAMA/CLX¯ (0.59 g, 0.97 mmol) and MMA (0.3 mL, 2.90 mmol), MeOH (0.59 mL), THF (0.59 mL), bpy (6.1 mg, 0.039 mmol), and MI with 29% of bromoester groups (20.3 mg, including 0.039 mmol of initiating sites) were placed into a Schlenk flask and degassed by three freeze–pump–thaw cycles. The initial sample was taken and the CuCl catalyst (2.9 mg, 0.019 mmol) was introduced to the mixture. The reaction was carried out for 2 h and then stopped by exposing to air. The catalyst was removed by passing the solution through the alumina column. The polymer was precipitated in THF-diethyl ether mixture and then dried under the vacuum.

#### 3.2.2. Ionic Graft Copolymers Bearing FUS¯

The comonomers TMAMA/FUS¯ (0.43 g, 0.63 mmol) and MMA (0.2 mL, 1.90 mmol), MeOH (0.86 mL), THF (0.86 mL), bpy (3.9 mg, 0.025 mmol), and MI with 29% of bromoester groups (13.1 mg, including 0.025 mmol of initiating sites) were placed into a Schlenk flask and degassed by three freeze–pump–thaw cycles. The initial sample was taken and the CuCl catalyst (1.9 mg, 0.013 mmol) was introduced to the mixture. The reaction was carried out for 4 h and then stopped by exposing to air. Further steps of the procedure were the same as the above described for the copolymer with CLX anions.

#### 3.2.3. Ionic Graft Copolymers Bearing CLX¯ and FUS¯

The comonomers TMAMA/CLX¯ (0.22 g, 0.36 mmol), TMAMA/FUS*¯* (0.25 g, 0.36 mmol), and MMA (0.23 mL, 2.20 mmol), MeOH (0.94 mL), THF (0.94 mL), bpy (4.5 mg, 0.029 mmol), and MI with 29% of bromoester groups (15.0 mg, including 0.029 mmol of initiating sites) were placed into a Schlenk flask and degassed by three freeze–pump–thaw cycles. The initial sample was taken and the CuCl catalyst (2.2 mg, 0.014 mmol) was introduced to the mixture. The reaction was carried out for 4 h and then stopped by exposing to air. Further steps of procedure were as the same as for the above copolymers.

^1^H NMR data for side chains of polymers (DMSO-d_6_, ppm): 0.63–0.84 ppm (3H, -CH_3_), 1.83–1.97 ppm (2H, -CH_2_-), 3.1–3.27 ppm (9H, -N^+^(CH_3_)_3_), 3.51–3.62 ppm (3H, -O-CH_3_), 3.64–3.78 ppm (2H, -CH_2_-N^+^-), and 4.4–4.6 ppm (2H, -CH_2_-O-).

FT-IR data (Figure S2): C-N^+^ and C-H (2860–2950 cm^−1^ and 1440–1490 cm^−1^), C=O (1726–1717 cm^−1^), C-N (1635 cm^−1^), and C-O ester bonds (1130–1200 cm^−1^). CLX¯: C=O (1715 cm^−1^), C=C aromatic ring (1610 and 1470 cm^−1^), C-N (1300–1320 cm^−1^), C=C (3000 cm^−1^), C-Cl (750 cm^−1^), benzene derivative (708 cm^−1^). FUS¯: =C< (1360–1380 cm^−1^), C=C at (, 1550 cm^−1^, 3030 cm^−1^), C-H (2860–2930 cm^−1^), and trisubstituted alkene (840 cm^−1^).

### 3.3. Release Studies of Ionic Drugs

Copolymers containing bioactive anions were dissolved in PBS (pH = 7.4) to obtain 1 mg/mL solutions. Then, 1 mL of the obtained solution was poured into a dialysis membrane bag (MWCO = 3.5 kDa). After that, the membrane was drowned in a glass vial filled with 44 mL of PBS and stirred at 37 °C. Then, 0.5 mL portions of samples were taken during drug release and mixed with 0.5 mL of MeOH. The probes were analyzed by UV–Vis, observing the absorption local maxima at λ = 207 and 220 nm for CLX¯ and λ = 207 nm for FUS¯. The drug content in the ionic conjugates (DC) and amount of released drug (ARD) were calculated using the generated calibration curves and absorbance maxima at the proper wavelengths for the anionic drugs.

In the case of the dual drug system, the calculation of both individual drug contents (DC) was impossible because the signals of both drugs overlapped. To overcome this problem, a series of PBS solutions containing both drugs in the ratio of 1:1 wt/wt with concentrations ranging within 0.0425–0.0013 μg/mL were prepared in order to collect absorbance data and generate a new calibration curve. The curve was used to compute the total drug content of both FUS¯ and CLX¯. Since it was possible to receive DC of CLX¯ at λ = 220 nm, the DC of FUS¯ was calculated by subtracting these two values.

### 3.4. Cell Culture

Both types of normal cells, i.e., BEAS-2B and NHDF, were grown in a DMEM-F12 medium in sterile culture bottles (75 cm^2^ area) with 10% (*v/v*) FBS and 1% antibiotics (10,000 µg/mL of streptomycin and 10,000 units/mL of penicillin) at 37°C in the incubator (humidified atmosphere composed of 5% CO_2_). The cell cultures were set in a 96-well plate at a density of 8500 cells per well.

### 3.5. TT Cytotoxicity Test

First, 8500 cells were placed into 96-well plates in 0.2 mL medium and incubated overnight. Untreaded (control) cells were located in the first row and outer columns of wells. A batch of dilutions containing suspended polymer systems (3.125–100 µg/mL) were placed in the remaining wells. The treated and control cells were incubated for 72 h in standard conditions. After this time, the polymer solutions were disposed of and 50 µL of MTT solution (0.5 mg/mL in RPMI 1640 without phenol red) was placed into each well. After the generation of formazan crystals (1–2 h of incubation), the MTT solution was taken out of the titer plates and crystals were dissolved in 75 µL of isopropanol/HCl solution (*v/v* 1/0.04). Next, the absorbance of the formazan product was measured at 570 nm using a microplate reader. Three independent tests were performed for each cell line and readings were repeated three times for each concentration. The cytotoxicity was measured by relative cell viability evaluated as the percentage fraction of the control cells (100%).

### 3.6. Characterization

^1^H NMR spectra were registered by a UNITY/NOVA spectrometer (Varian, Mulgrave, Victoria, Australia) operating at 300 MHz. The measurements were performed for the samples in deuterated dimethyl sulfoxide (DMSO-d6) with tetramethylsilane (TMS) as an internal standard. Molecular weight and dispersity index (M_n_ and Ð) were estimated by gel permeation chromatography (GPC). Measurements were performed on an Ultimate 3000 chromatograph (Thermo Fisher Scientific, Waltham, MA, USA) with a differential refractometer RefractoMax 521 detector. The polymer samples, prepared in DMF containing 10 mM LiBr at 50 °C, were passed through a TSKgel Guard SuperMPHZ-H 6 µm pre-column (4.6 mm × 2 cm) and two TSKgel SuperMultiporeHZ-H 6 µm columns (4.6 mm × 15 cm) with a flow rate of 0.25 mL/min. The calculations were based on poly(ethylene oxide) (PEO) standards (982–969,000 g/mol). Fourier transform infrared (FT-IR) spectroscopy was conducted with a Spectrum Two 1000 FT-IR Infrared spectrometer (Perkin Elmer, Waltham, MA, USA) with attenuated total reflection (ATR). During drug release, the samples taken in appropriate times intervals were analyzed by ultraviolet−visible light spectroscopy (UV−Vis, spectrometer Evolution 300, Thermo Fisher Scientific, Waltham, MA, USA) in quartz cells to determine the drug content (DC) and the amount of released pharmaceutical anions. Viability monitoring was performed using Live Cell Analyzer (JuLI™ Br; NanoEnTek Inc., Seoul, Korea). The cytotoxicity was evaluated by measuring the absorbance utilizing a microplate reader (Epoch, BioTek, Winooski, VT, USA).

## 4. Conclusions

The biologically active TMAMA/Cl as the polymerizable IL-containing choline unit was successfully biofunctionalized by the introduction of CLX¯ and FUS¯ as the pharmaceutical anions with antibacterial activity via the ion exchange process. After modification, the choline-based IL monomers were used in controlled polymerization to prepare the well-defined graft copolymers with different contents of IL units (19–50 mol%) in the side chains. These drug conjugates carrying one or two types of drug anion(s), i.e., CLX¯ and/or FUS¯ (41–44 and 33–53%, respectively) were designed to investigate their drug release properties. The kinetics profiles of in vitro drug release demonstrated two regions, the burst effect (up to 1 h for 15% of drug) and then the plateau-like effect (up to 50 h). The amounts of released CLX¯ and FUS¯ reached 21–24% (2.2–2.7 μg/mL) and 23–27% (2.0–3.6 μg/mL), respectively, and represented no significant influence of the pharmaceutical anion type on the delivery effectiveness of the studied polymer conjugate systems. In addition, the biological potential of the conjugates was evaluated against NHDF and BEAS-B human normal cells, indicating low cytotoxicity and resulting tissue biocompatibility. It was observed that, in general, with an exception for G_CLX¯ at 100 µg/mL, the polymer concentrations of 3 and 100 µg/mL did not lower or greatly increase cells confluency, which additionally confirmed the low toxicity of the tested carriers. Generally, direct preparation of ionic drug conjugates from pharmaceutically functionalized choline IL monomers seems to be a convenient strategy leading to innovative graft polymers bearing CLX¯ and/or FUS¯ anions as promising materials for drug delivery or co-delivery systems.

## Figures and Tables

**Table 1:** Characteristics of graft copolymers carrying pharmaceutic anions.

No.	Time (h)	X_TMAMA_^a^ [%]	DP_TMAMA_^a^	DP_sc_^a^	F_TMAMA_^a^ [mol %]	M_n_^a^·10^−3^ [g/mol]	M_n_^b^·10^−3^ [g/mol]	Ð ^b^
G_Cl [26]	2	30	8	35	39	273.1	12.5	1.15
G_CLX¯	2	33	8	43	19	433.6	17.4	1.4
G_FUS¯	4	50	13	26	50	517.1	18.5	1.5
G_CLX¯/FUS¯	4	24	3/3	21	29	283.5	11.7	1.9

Conditions: G_CLX¯ [TMAMA/CLX¯]_0_:[MMA]_0_:[MI]_0_:[CuCl]_0_:[bpy]_0_ = 25:75:1:0.5:1, methanol:THF = 1:1 *v/v*, where methanol:TMAMA/CLX¯ = 1:1 *v/wt*, G_FUS¯ [TMAMA/FUS¯]_0_:[MMA]_0_:[MI]_0_:[CuCl]_0_:[bpy]_0_ = 25:75:1:0.5:1, MeOH:THF = 1:1 *v/v*, where MeOH:TMAMA/FUS¯ = 2:1 *v/wt*, G_CLX¯/FUS¯ [TMAMA/CLX¯]_0_:[TMAMA/FUS¯]_0_:[MMA]_0_:[MI]_0_:[CuCl]_0_:[bpy]_0_ = 12.5:12.5:75:1:0.5:1, MeOH:THF = 1:1 *v/v*, where MeOH:TMAMA/FUS¯:TMAMA/CLX¯ = 2:1:1 *v/wt/wt*, 40 °C, MI: MMA/BIEM = 71/29; DP_n_ = 165, DP_BIEM_ = n_sc_ = 48, ^a^ by ^1^H NMR, where X_TMAMA_ TMAMA conversion, DP_sc_ - polymerization degree of side chains, F_TMAMA_ - content of TMAMA in copolymer, ^b^ by SEC (DMF, PEO calibration).

**Table 2:** Data for drugs released from polymeric carriers.

No.	Drug	DC (%)	CRD (μg/mL)	ARD (%)
G_CLX¯	CLX¯	44	2.7	24
G_FUS¯	FUS¯	53	3.6	27
G_CLX¯/FUS¯	CLX¯	41	2.2	21
	FUS¯	33	2.0	23

Where: DC is drug content, CRD is concentration of released drug, ARD is amount of released drug.

## Data Availability

Not applicable.

## Supplementary Materials

The following supporting information can be downloaded at: https://www.mdpi.com/article/10.3390/ijms232314731/s1.
