# Supplementary material for: Pharmaceutical Functionalization of Monomeric Ionic Liquid for the Preparation of Ionic Graft Polymer Conjugates
Source: Int J Mol Sci. 2022 Nov 25;23(23):14731. doi: 10.3390/ijms232314731 (PMC9735495; doi:10.3390/ijms232314731)
Supplement: Supplementary file 1 [file ijms-23-14731-s001.zip › ijms-2007296-supplementary.pdf]

Supplementary Materials

Pharmaceutical functionalization of monomeric ionic liquid for the preparation of ionic graft polymer conjugates

Aleksy Mazur, Katarzyna Niesyto, Dorota Neugebauer*

Department of Physical Chemistry and Technology of Polymers, Faculty of Chemistry, Silesian University of Technology, 44-100 Gliwice, Poland;

* Correspondence: dorota.neugebauer@polsl.pl

Contents:

Figure S1. IR spectra of pharmaceutically functionalized monomeric IL.

Figure S2. FT-IR spectra of TMAMA based copolymers.

Figure S3. Fragments of ^{1}H NMR spectra with signals of trimethylammonium groups in IL monomers before and after ion exchange.

Figure S4. ^{1}H NMR spectrum of G_CLX^{-}/FUS^{-} copolymer after purification.

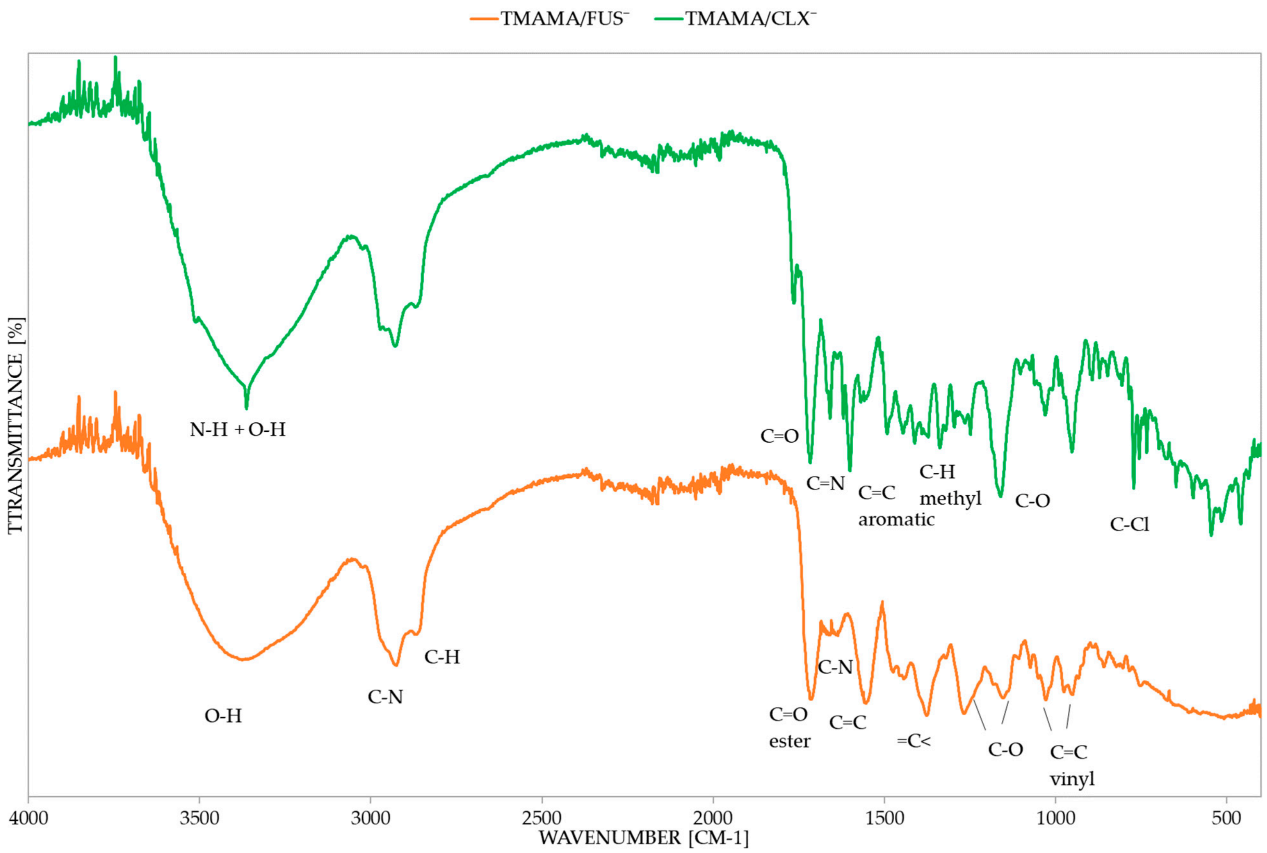

Figure S1. IR spectra of pharmaceutically functionalized monomeric IL.

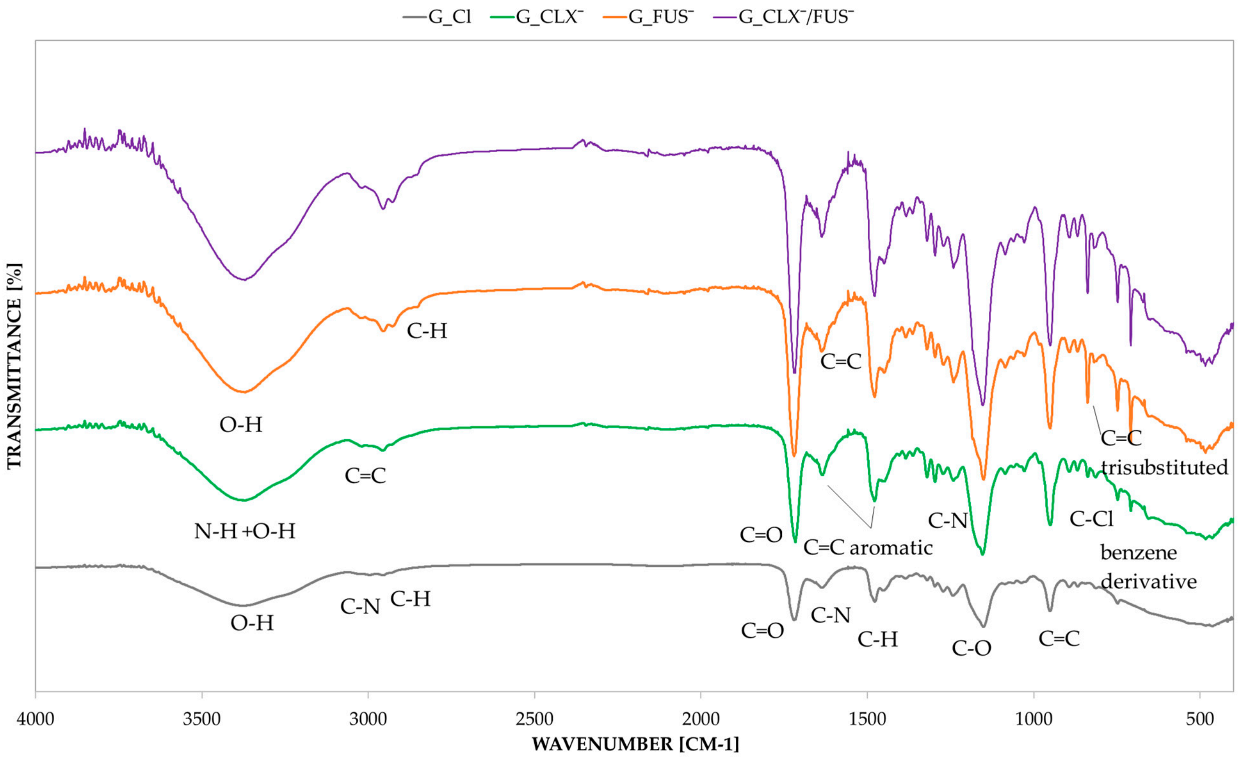

Figure S2. FT-IR spectra of TMAMA based copolymers.

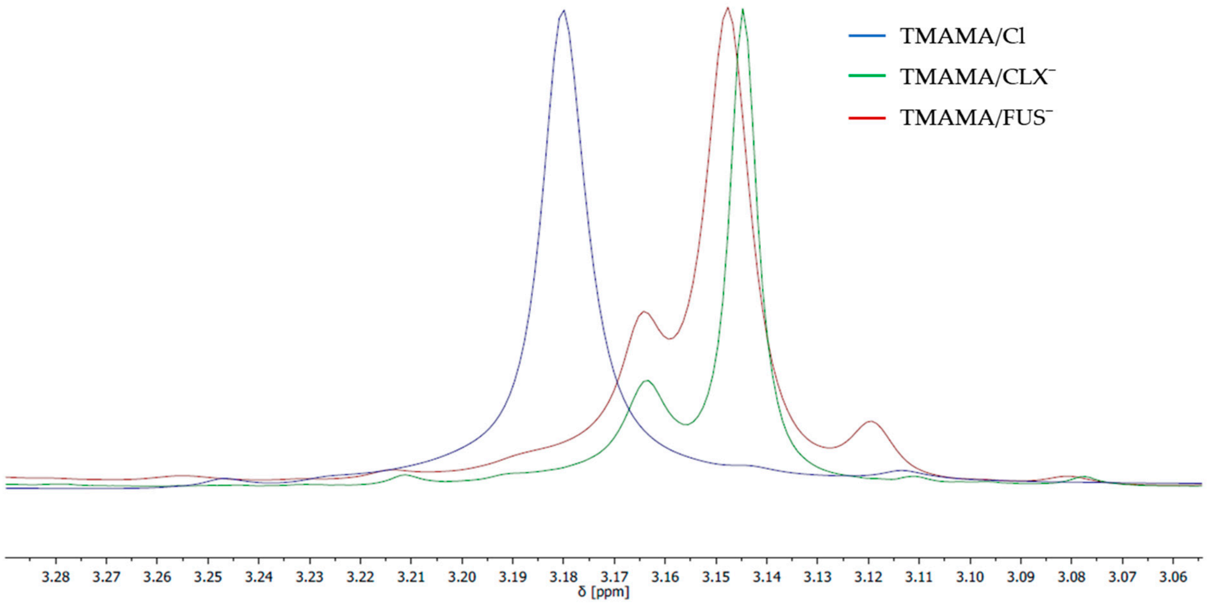

Figure S3. Fragments of ^{1}H NMR spectra with signals of trimethylammonium groups in IL monomers before and after ion exchange.

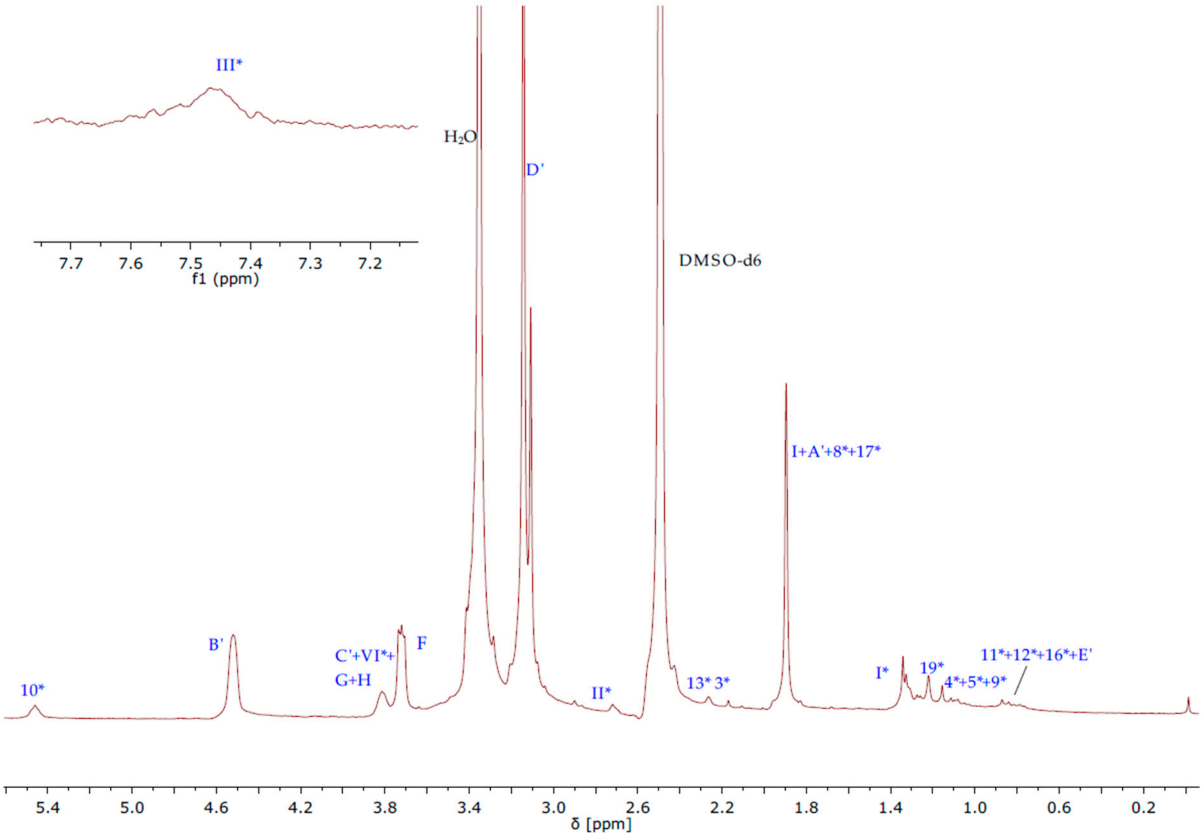

Figure S4. ^{1}H NMR spectrum of G_CLX^{-}/FUS^{-} copolymer after purification where the signals coming from pharmaceutical anions are denoted using the symbol *.
